# Microgeographic population structuring in a genus of California trapdoor spiders and discovery of an enigmatic new species (Euctenizidae: *Promyrmekiaphila korematsui* sp. nov.)

**DOI:** 10.1002/ece3.10983

**Published:** 2024-03-01

**Authors:** James Starrett, Emma E. Jochim, Iris L. Quayle, Xavier J. Zahnle, Jason E. Bond

**Affiliations:** ^1^ University of California, Davis Davis California USA

**Keywords:** Araneae, gene flow, machine learning, speciation, species delimitation, ultraconserved elements

## Abstract

The recognition and delineation of cryptic species remains a perplexing problem in systematics, evolution, and species delimitation. Once recognized as such, cryptic species complexes provide fertile ground for studying genetic divergence within the context of phenotypic and ecological divergence (or lack thereof). Herein we document the discovery of a new cryptic species of trapdoor spider, *Promyrmekiaphila korematsui* sp. nov. Using subgenomic data obtained via target enrichment, we document the phylogeography of the California endemic genus *Promyrmekiaphila* and its constituent species, which also includes *P. clathrata* and *P. winnemem*. Based on these data we show a pattern of strong geographic structuring among populations but cannot entirely discount recent gene flow among populations that are parapatric, particularly for deeply diverged lineages within *P. clathrata*. The genetic data, in addition to revealing a new undescribed species, also allude to a pattern of potential phenotypic differentiation where species likely come into close contact. Alternatively, phenotypic cohesion among genetically divergent *P. clathrata* lineages suggests that some level of gene flow is ongoing or occurred in the recent past. Despite considerable field collection efforts over many years, additional sampling in potential zones of contact for both species and lineages is needed to completely resolve the dynamics of divergence in *Promyrmekiaphila* at the population–species interface.

## Graphical abstract

Cryptic species, those that are difficult to distinguish using morphological characters, remain a perplexing problem in systematics and evolution. We use phylogenomic, population genomic, and morphological approaches and document the discovery of a new cryptic species of trapdoor spider in the California endemic genus *Promyrmekiaphila*.

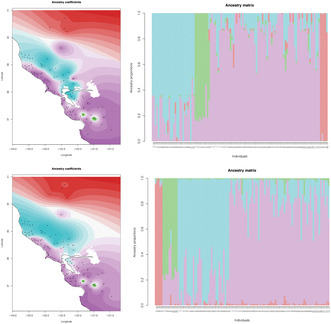

## 1 INTRODUCTION

Evaluating speciation mechanisms in cryptic species is a multifaceted challenge. Cryptic species are typically morphologically conserved in traits used in species determination (i.e., diagnostic taxonomic characters); thus, one must establish, via other lines of evidence, that speciation has indeed occurred before exploring nuanced questions regarding causation. Cryptic species often entail morphologically homogenous lineages that are divergent genetically; that is, genetic divergence often greater than what is observed in taxa that display phenotypic differences (Bickford et al., 2007; Struck et al., 2018). Such marked genetic divergence is often attributed to factors like low vagility, genetic drift, natural selection (or lack thereof), and niche conservatism (Wiens, 2004). Moreover, when species remain in allopatry, reinforcement mechanisms (e.g., character displacement) are less likely, thus increasing the likelihood that phenotypic similarity (crypsis) is maintained (Servedio & Noor, 2003). Organismal groups comprising divergent cryptic species that contain combinations of allopatric and parapatric/sympatric populations have the potential to illuminate speciation process in such systems.

Cryptic species have long been hypothesized to account for an underestimation of diversity in many groups of mygalomorph spiders (Bond et al., 2001; Hedin et al., 2015; Satler et al., 2013). The spider infraorder Mygalomorphae (tarantulas, trapdoor spiders, and their kin) is composed of species that are generally long‐lived, exhibit high morphological conservation, and have simple genitalic structures. These spiders live in subterranean silk‐lined burrows or funnel/sheet webs under large debris or in crevices, consistent with their fossorial morphology (Wilson et al., 2023). Mygalomorphs exhibit low vagility and intense connectivity to the landscape; individuals typically begin excavating a new burrow as an early instar and inhabit that burrow for their entire lifespan (Bond & Coyle, 1995). Genetic data have been used to show lineage divergence arising due to geologic events (Hedin et al., 2013; Hendrixson & Bond, 2007; Opatova et al., 2013, 2016; Satler et al., 2013) and changes in climate (Hamilton et al., 2011; Huey et al., 2019; Řezáč et al., 2007; Rix, Cooper, et al., 2017), as well as extinction due to recent anthropogenic landscape change (Bond et al., 2006; Ferretti et al., 2018; Řezáč & Heneberg, 2014; Rix, Huey, et al., 2017). However, the mechanisms underlying pre‐ and post‐zygotic reproductive isolation in mygalomorphs are poorly understood.

The California endemic mygalomorph genus *Promyrmekiaphila* Schenkel 1950 comprises two nominal species, *P*. *clathrata* (Simon 1891) and *P*. *winnemem* (Stockman & Bond, 2008), that are allopatrically distributed. *Promyrmekiaphila clathrata* has been collected extensively throughout the coastal ranges in central and northern California and *P*. *winnemem* is known from the northern end of the Central Valley (Stockman & Bond, 2008). In a phylogeographic study based on mitochondrial DNA markers, Stockman and Bond (2007) documented a pattern of “extreme” genetic divergence coupled with high geographic concordance across most lineages. Their study identified *P. winnemem* as a distinct lineage but also recognized divergence between population groups within the *P. clathrata* lineage. Based on phylogenetic/genetic analyses of *P. clathrata* coupled with evaluations of demographic interchangeability applying Templeton's Cohesion Species Concept (CSC; Templeton, 1989), Stockman and Bond hypothesized that the lineage comprised 4–5 cryptic species. In a subsequent taxonomic review of the genus, Stockman and Bond (2008) described *P. winnemem* but maintained *P. clathrata* as a single species. Stockman and Bond (2007, 2008) also recognized the potential shortcomings of using only mtDNA to delimit species and thus were hesitant to describe additional species based on those data alone.

In this study, we generate sub‐genomic scale data to investigate phylogenetic relationships and population structure in *Promyrmekiaphila* and explore deep phylogenetic breaks hypothesized to occur across the genus. We employ multiple approaches to test species delimitation hypotheses and test for gene flow to assess reproductive isolation in divergent lineages. We measure an array of female somatic morphological characters to evaluate if species diverge morphologically in potential sympatry, as well as in allopatry with divergent ecological niches. Our goal is to address at what point in the speciation process morphologically conserved taxa can be declared cryptic species rather than simply divergent population groups. We describe a new species *Promyrmekiaphila korematsui* sp. nov. Ultimately, we reject dividing the most geographically widespread species, *P*. *clathrata*, into multiple species, and instead interpret genetic breaks as deeply structured populations that are not fully reproductively isolated or may have undergone historical gene flow.

## 2 MATERIALS AND METHODS

### 2.1 Sampling

We sampled from throughout the known distribution of *Promyrmekiaphila* and utilized specimens from Stockman and Bond (2008), as well as new records (Appendix S1). One to three individuals per geographic locality were sequenced. Individuals of *Aptostichus hedinorum*, *A. barackobamai*, *A*. *simus*, *A*. *stanfordianus*, and *Apomastus kristenae* were used as outgroups.

### 2.2 Sequence capture

Ultraconserved element (UCE) data were generated following the methods described in Faircloth et al. (2012) and further modified in Hedin et al. (2019), Kulkarni et al. (2020), and Starrett et al. (2017). Genomic DNA was extracted from leg tissue using the Blood and Tissue DNeasy kit (Qiagen) and quantified using Qubit. Two hundred and fifty nanograms of DNA was fragmented to 200–1000 bp using ultrasonication (Covaris). Libraries were generated with the KAPA Hyperprep kit (Roche) with universal adapters and iTru5/7 barcodes (Glenn et al., 2019) (BadDNA@UGA) and then hybridized at 60°C for 24 h to the Spider probeset (Kulkarni et al., 2020) following the version 4 chemistry protocol (Arbor Biosciences). Hybridization enriched library pools were sequenced with 150 bp paired‐end reads on the HiSeq4K at the University of California Davis DNA Technologies Core. Additional samples were processed by RAPID Genomics (Gainesville, FL).

Read processing and analyses were conducted on the University of California Farm Bioinformatics Cluster. Reads were filtered and trimmed using Illumiprocessor (Faircloth, 2013) and Trimmomatic (Bolger et al., 2014) in the Phyluce v.1.7.1 pipeline (Faircloth, 2016). Assemblies were conducted de novo with the cleaned paired‐end and single‐end reads using SPAdes v.3.14.1 with the isolate option (Prjibelski et al., 2020). Scaffolds were matched with 65% identity and 65% coverage to the modified probe list from (Maddison et al., 2020), which is a blend of the Arachnid (Faircloth, 2017; Starrett et al., 2017) and Spider (Kulkarni et al., 2020) probesets. These modified probes retain the spider probe sequences for contig‐matching in the case where the Kulkarni et al. (2020) set also contains arachnid probe sequences that match the same homolog (Maddison et al., 2020). MAFFT was used to align individual locus datasets (Katoh & Standley, 2013), and alignments with locus occupancy minimums of 95% and 75% were obtained. Alignment masking was performed with trimAl v.1.2 (Capella‐Gutierrez et al., 2009) using the strict, strictplus, and gappy options.

Cytochrome oxidase I (COI) sequences were sampled for individuals from a divergent clade (this study, see Section 3) that were not included in Stockman and Bond (2007), but see below. Clean read pairs were mapped to *P*. *clathrata* COI sequence EF543032 (Stockman & Bond, 2007) using Burrows‐Wheeler Aligner (BWA; Li & Durbin, 2009) with mismatch penalties of 2 and 4, and output of unpaired reads allowed. Alignments were evaluated in Geneious 10.23 (www.geneious.com) and consensus sequences were generated. To reduce gaps due to potential reference mismatch the most complete COI sequence (MY3285) was used as a reference for an additional round of mapping with BWA (mismatch penalty 2, output of unpaired reads allowed). Consensus sequences were generated in Geneious with ambiguities resolved with 90% majority‐rule.

### 2.3 Phylogenetic analyses

Phylogenetic trees were inferred from concatenated alignments generated from the minimum locus occupancy and alignment masking datasets using IQ‐TREE v.2.1.2 (Nguyen et al., 2015). The ModelFinder Plus option was used to determine the best model for each partition (Kalyaanamoorthy et al., 2017), and ultrafast bootstrapping was performed with 1000 pseudoreplicates (Hoang et al., 2018). Individual locus gene trees were inferred using IQ‐TREE for the minimum 95% and 75% occupancy loci and used to generate a coalescent based tree with ASTRAL‐III (Zhang et al., 2018). Multispecies coalescent (MSC) bootstrapping was run with ASTRAL v.5.7.4 and 100 pseudoreplicates (Simmons et al., 2019). Hypothesized lineages for species delimitation analyses (see below) were based on the strict consensus tree from the MSC bootstrap analysis of the 95% occupancy gene trees, with initial groups based on recovered nodes and with single terminals incorporated when sister to the recovered group.

For COI, consensus sequences from BWA were aligned unambiguously with *Promyrmekiaphila* sequences from Stockman and Bond (2007), including euctenizid outgroups *Eucteniza cabowabo* (KY017710) and *Neoapachella rothi* (KY017712). Phylogenetic analysis was conducted with IQ‐TREE v.2.1.2 (Nguyen et al., 2015). Data were partitioned by codon position with the GTR + G model used for each partition. Ultrafast bootstrapping was performed with 1000 pseudoreplicates (Hoang et al., 2018).

### 2.4 Species delimitation analyses

#### 2.4.1 Variational autoencoder clustering

We generated SNP datasets for genetic based cluster analyses. Reads were mapped against corresponding scaffolds with BWA (Li & Durbin, 2009) and phased alignments were generated within the Phyluce pipeline. Independent phased alignment datasets were generated for all *Promyrmekiaphila* specimens (referred to as ALL), and *P*. *clathrata* individuals only (see Section 3). We generated minimum locus occupancy datasets of 95% and 75% for both the ALL and *P*. *clathrata* only datasets. Phased alignments were screened for SNPs; five sets of single random SNP per locus (i.e., unlinked SNPs) datasets were generated to test for SNP set sensitivity. SNP sets were converted to one‐hot encoding and applied to an unsupervised clustering analysis that uses variational auto encoders (VAE) (Derkarabetian et al., 2019). The distribution of latent variables inferred by the encoder is given as a normal distribution with mean and standard deviation for each SNP set based on 100 replicates, which is then decoded and plotted in a two‐dimensional graph.

#### 2.4.2 Ancestry coefficient analysis and gene flow tests

The SNP sets (detailed above) were also used to generate ancestry coefficient (*Q*‐matrix) maps and ancestry proportion barplots. SNP datasets in structure format were converted to lfmm format using the R package LEA (Frichot & François, 2015). Tess3r v1.1.0 was used to compute *Q*‐matrices based on different values of *K*, or the number of ancestral populations (Caye et al., 2016). The default value was used for the regularization parameter lambda. For the ALL SNP datasets, analyses were run with a *K* value of 4 to determine if individuals were assigned to the four major clades within *Promyrmekiaphila* (i.e., *P*. *korematsui* sp. nov., *P*. *winnemem*, *P*. *clathrata* α, and *P*. *clathrata* β; see Section 3). For the *P*. *clathrata* only datasets, analyses were run with a *K* value of 11 to determine if individuals were assigned to the hypothesized lineages from the MSC bootstrap (i.e., *P*. *clathrata* C‐M), and *K* = 2 to determine if individuals were assigned to the major phylogenetic split within *P*. *clathrata* (i.e., *P*. *clathrata* α and β). Barplots were generated showing the ancestry proportion for each individual under the different *K* value scenarios. We used marmap v.1.0.6 (Pante & Simon‐Bouhet, 2013) to import bathymetric data at a resolution of 1 min, clipped to the distribution of *Promyrmekiaphila* localities (ALL: latitude: 35.5–41.0, longitude: −124.5 to −120.5; *P*. *clathrata* only: latitude: 35.5–40.0, longitude −124.5 to −120.5), from the ETOPO1 (Amante & Eakins, 2009) database on the NOAA server. These data were converted to a raster using marmap. The ancestry coefficients from tess3r were plotted on the map for all individuals, with fit of interpolating surfaces for irregular shaped data calculated using the Fields Krig Model with theta set at 10.

To test if gene flow has occurred between *P*. *korematsui* sp. nov. and *P*. *clathrata*, we calculated the D3 statistic (Hahn & Hibbins, 2019). The D3 statistic ranges from −1 to 1, with a value of 0 expected under an incomplete lineage sorting only scenario. Pairwise‐differences among concatenated UCE loci (minimum 95% locus occupancy), corrected with the HKY model and neighbor‐joining tree, were generated using Geneious Tree Builder (Geneious Prime 2023.2.1) for all individuals from *P*. *korematsui* sp. nov., *P*. *clathrata*, and *P*. *winnemem*. Average pairwise distances among the three species were calculated.

We tested for gene flow between the two major *P*. *clathrata* clades (α and β) using 3s (Dalquen et al., 2017). We selected two groups of one individual each of *P*. *clathrata* α and *P*. *clathrata* β, and for outgroup, *P*. *winnemem*. The two groups differed in the proximity of the *P*. *clathrata* α and β individuals to each other (Group 1: 85.3 km; Group 2: 4.7 km). One hundred percent locus occupancy datasets were aligned using MAFFT and filtered with trimAl, as implemented in Phyluce. The M0 (no gene flow) and M2 (isolation with migration, asymmetrical) models in 3s were compared using a likelihood ratio test with significance determined with two degrees of freedom. Each analysis was run three times to evaluate consistency. A Gaussian quadrature with *K* = 16 points was used for integrating over coalescent times in the gene trees. Marginal likelihood estimates (MLE) for M2 are reported for ancestral population size (*θ*_(αβw)_ and *θ*_(αβ)_), divergence times (*τ*_(αβw)_ and *τ*_(αβ)_), current population sizes (*θ*_(α)_ and *θ*_(β)_), and the expected number of migrant individuals from one population to another per generation (*M*_(αβ)_ and *M*_(βα)_).

#### 2.4.3 Supervised machine learning classification

We used the supervised machine learning program CLADES (Pei et al., 2018) to further test species limits within *P*. *clathrata* (see Section 3). Minimum 95% locus occupancy datasets were generated using Phyluce for all *P*. *clathrata* individuals as well as two separate datasets representing the groups formed by the major phylogenetic split within *P*. *clathrata* (see Section 3). Delimitation analyses were performed using two training models of genetic characteristics of species: the default ALL_CLADES model, which was generated from simulations of a two‐species model based on a broad range of taxa (see Pei et al., 2018 for details), and the Metano_CLADES model from Derkarabetian et al. (2022), which was generated based on data from a short range endemic arachnid genus (*Metanonychus*) that has similar natural history characteristics to mygalomorph spiders.

#### 2.4.4 Morphological analyses

We examined 81 adult females under a dissecting scope and measured 17 continuous somatic characters following Bond (2012). Males are rarely collected and thus an insufficient sample size was obtained for statistical analysis of measurement data taken from males. Measurements were transformed to log normal values, and a principal component analysis was conducted using the ggplot2 v.3.3.6 package (Wickham, 2016) in R (R Core Team), following Hamilton et al. (2016). Individuals were assigned to the four hypothesized species for visualization (*P. korematsui* sp. nov., *P. winnemem*, *P. clathrata* α, *P. clathrata* β). Ellipses for groups represent the multivariate *t*‐distribution at the 95% confidence level.

We further investigated morphological separation among the four hypothesized species by conducting a linear discriminant analysis (LDA) with cross validation with the MASS v.7.3‐51.5 package (Venables & Ripley, 2002) in R. Data were split into training/testing proportions of 0.8/0.2 using the caret v.6.0‐93 package (Kuhn, 2008). Jitter plots were generated for each character using the tidyverse v.0.9‐2 package (Wickham et al., 2019) in R and assessed for overlap.

#### 2.4.5 Taxonomic abbreviations and description

BME (Bohart Museum of Entomology; Davis, California) and CAS (California Academy of Sciences; San Francisco, California).

Quantitative Morphological Abbreviations (defined and illustrated in Bond, 2012).

ANTd: number of teeth on the anterior margin of cheliceral fang furrow.
Cl, Cw: carapace length and width. Carapace length taken along the midline dorsal‐most posterior position to the anterior front edge of the carapace (chelicerae are not included in length). Carapace width taken at the widest point.
AME, ALE, PME, PLE: anterior median, anterior lateral, posterior median, and posterior lateral eyes, respectively.
LBl, LBw: labium length and width taken from the longest and widest points, respectively.
PTl, PTw: male palpal tibia length and width.
Bl: palpal bulb length from embolus tip to the bulb base, taken in the ventral plane at its longest point.
PTLs, TBs: number of female prolateral patella and tibial spines leg III.
STRl, STRw: sternum length and width. Sternum length from the base of the labium to its most posterior point. Width taken across the widest point, usually between legs II and III.
PLS: posterior lateral spinneret.
TSrd, TSp, TSr: number of tibial spines on the distal most retrolateral, prolateral, and midline retrolateral positions.
ITC: inferior tarsal claw.

##### Measurement, characterization, and illustration of morphological features

Description of morphological features evaluated follows Bond (2012). Unique voucher numbers were assigned to all specimens (alphanumeric designations beginning with BMEA); these data were added to each vial and can be used to cross‐reference all images, measurements, and locality data. All measurements are given in millimeters and were made with a Leica MC205 dissecting microscope equipped with the Leica Analysis Suite Software. Lengths of leg articles were taken from the mid‐proximal point of articulation to the mid‐distal point of the article (sensu Bond, 2012, figs 11–16). Leg I and Leg IV article measurements are listed in the species description in the following order: femur, patella, tibia, metatarsus, tarsus. Carapace and leg coloration are described semi‐quantitatively using Munsell® Color Charts (Windsor, NY) and are given using the color name and color notation (hue value/chroma).

Digital images of specimens were made using a BKPlus Digital Imaging System (Dun Inc.TM, Richmond, VA) where images were recorded at multiple focal planes and then assembled into a single focused image using the computer program Helicon Focus (Helicon Soft, Ltd., Ukraine). The female genital region was removed from the abdominal wall and tissues dissolved using trypsin; spermathecae were examined and photographed in the manner described above.

Latitude and longitude for the collecting locality was recorded in the field using a Garmin® Global Positioning System receiver (Garmin International Ltd., Olathe, KS) using WGS84 map datum.

## 3 RESULTS

### 3.1 Data summary

Detailed locality information regarding sampling is provided in Appendix S1. A total of 115 *Promyrmekiaphila* individuals and seven outgroup individuals were processed for sequencing of UCE data. Detailed information regarding sequencing results, assemblies, scaffold match, and alignment are provided in DRYAD (Starrett et al., 2024: https://doi.org/10.5061/dryad.4mw6m90gv). Average read count per individual obtained was 3,678,012 and ranged from 66,984 to 8,427,177. Average number of probe matched scaffolds per individual obtained was 1297 and ranged from 503 to 1435. The locus occupancy minimums of 95% and 75% resulted in locus counts of 204 and 1235, respectively. Demultiplexed raw reads are available in SRA database (PRJNA1019074). Assembled contigs, sequence alignments, SNP sets, phylogenetic trees, pairwise genetic distances, morphological measurement data, and supplementary figures are available in DRYAD (https://doi.org/10.5061/dryad.4mw6m90gv).

### 3.2 *Promyrmekiaphila* phylogeny

**FIGURE 1:**
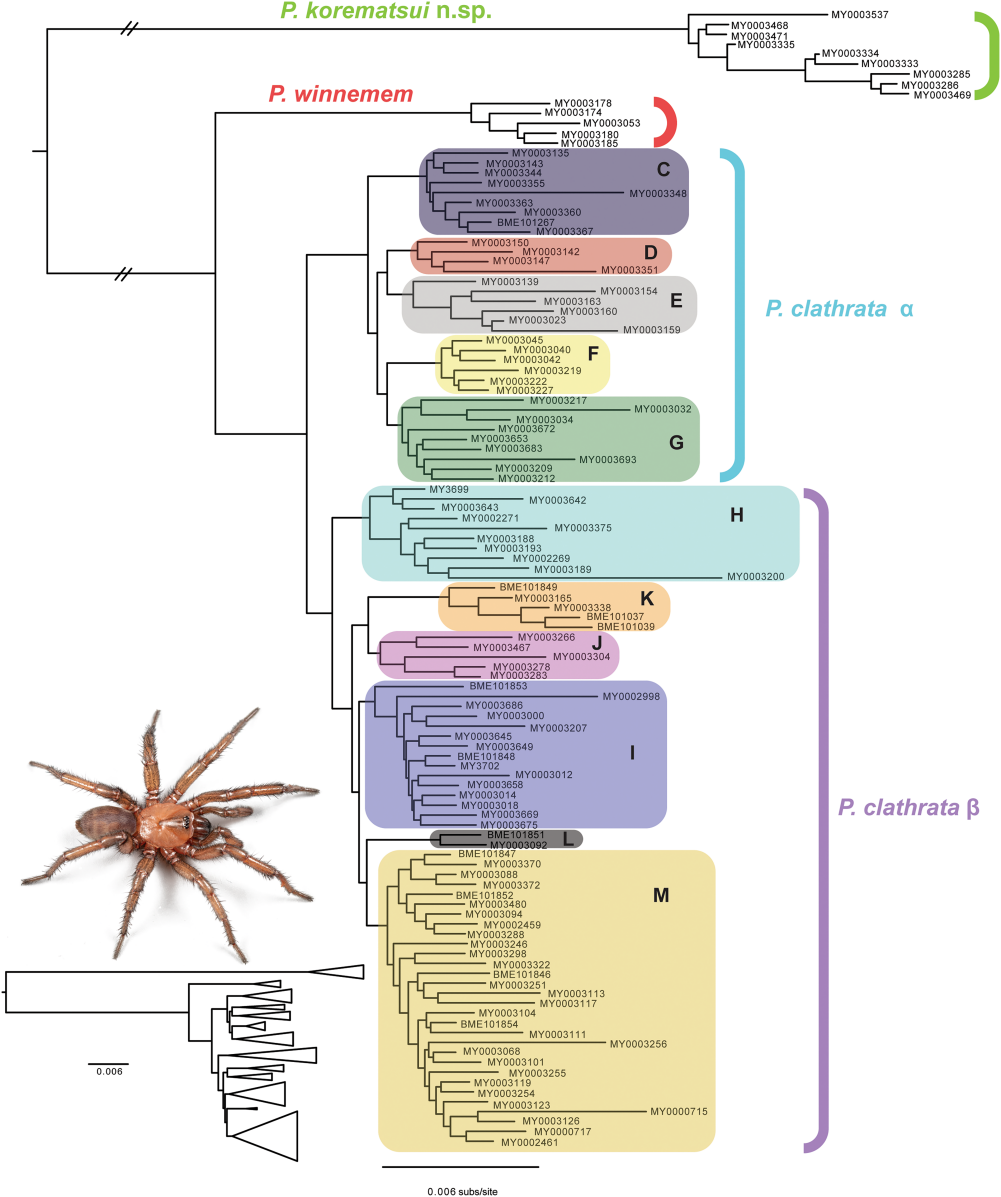
Maximum likelihood phylogram of *Promyrmekiaphila*, based on the minimum 95% locus occupancy concatenated dataset. Shaded box groups are based on the multi‐species coalescent ASTRAL bootstrap analysis (see Figure S2). Subset image shows *P. korematsui* sp. nov. HOLOTYPE male live habitus, dorsal view. Subset tree shows branch lengths among species and phylogeographic groups. See Figure S1 for tree with outgroups and bootstrap nodal support values.

Phylogenetic trees inferred from the concatenated UCE locus datasets from the different data masking settings and minimum occupancy thresholds were largely congruent, with differences occurring near the terminals of the tree (not shown). Thus, we focus on the trees based on the 95% minimum locus occupancy with strict data masking only (Figure 1; Figure S1). Coalescent‐based phylogenies were largely congruent with those based on concatenation, recovering the same major groups (Figure S2). Discrepancies in relationships among groups occurred at nodes with low support (posterior probability <.90 (not shown)).

**FIGURE 2:**
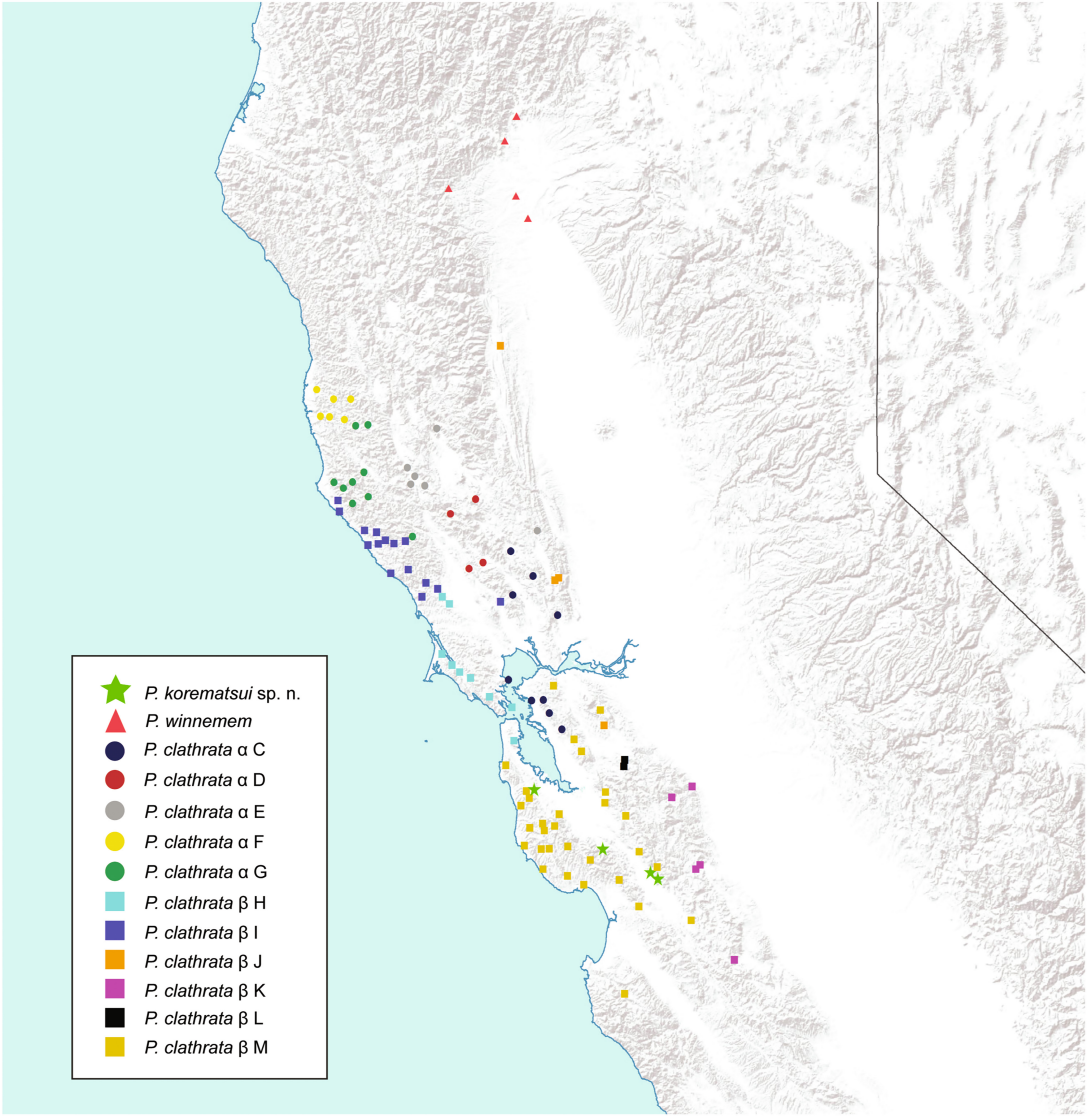
Distribution map of *Promyrmekiaphila* species and phylogeographic groups in California. Group colors are consistent with Figure 1.

Three major groups were recovered for *Promyrmekiaphila*, two of which correspond to *P. clathrata* and *P. winnemem*, which are sister taxa (Figure 1; Figures S1 and S2). This clade is sister to a third clade, which consists of individuals from four localities distributed roughly along the San Andreas Fault, adjacent to the eastern side of the Santa Cruz Mountains (Figure 2). This clade represents a previously unrecognized species of *Promyrmekiaphila* (*P. korematsui* sp. nov.). *Promyrmekiaphila clathrata* is considerably more widespread geographically than *P. winnemem* and *P. korematsui* sp. nov. and exhibits deep genetic structuring that comprises well‐supported phylogenetic/geographic groups recovered in both concatenated and coalescent based analyses. Eleven clades were recovered from the strict consensus of the MSC bootstrap analysis (Figure S2). We treated these clades, along with individual terminals combined (i.e., “lumped”) with their sister clades, as lineages that were tested as hypothesized species in downstream analyses (see below).

The COI phylogeny was similar to the trees based on the UCE data, supporting *P*. *korematsui* sp. nov. as sister to a clade of *P*. *winnemem* plus *P*. *clathrata* (Figure S3). The crown node for each of the three species has high bootstrap support (>94). Within *P*. *clathrata*, the α group is paraphyletic with respect to a monophyletic β group. Eight of the eleven *P*. *clathrata* lineages recognized by the UCE data were also recovered with the COI data. The α group E lineage is paraphyletic, and the β group H lineage is polyphyletic.

### 3.3 Species delimitation

#### 3.3.1 VAE clusters

**FIGURE 3:**
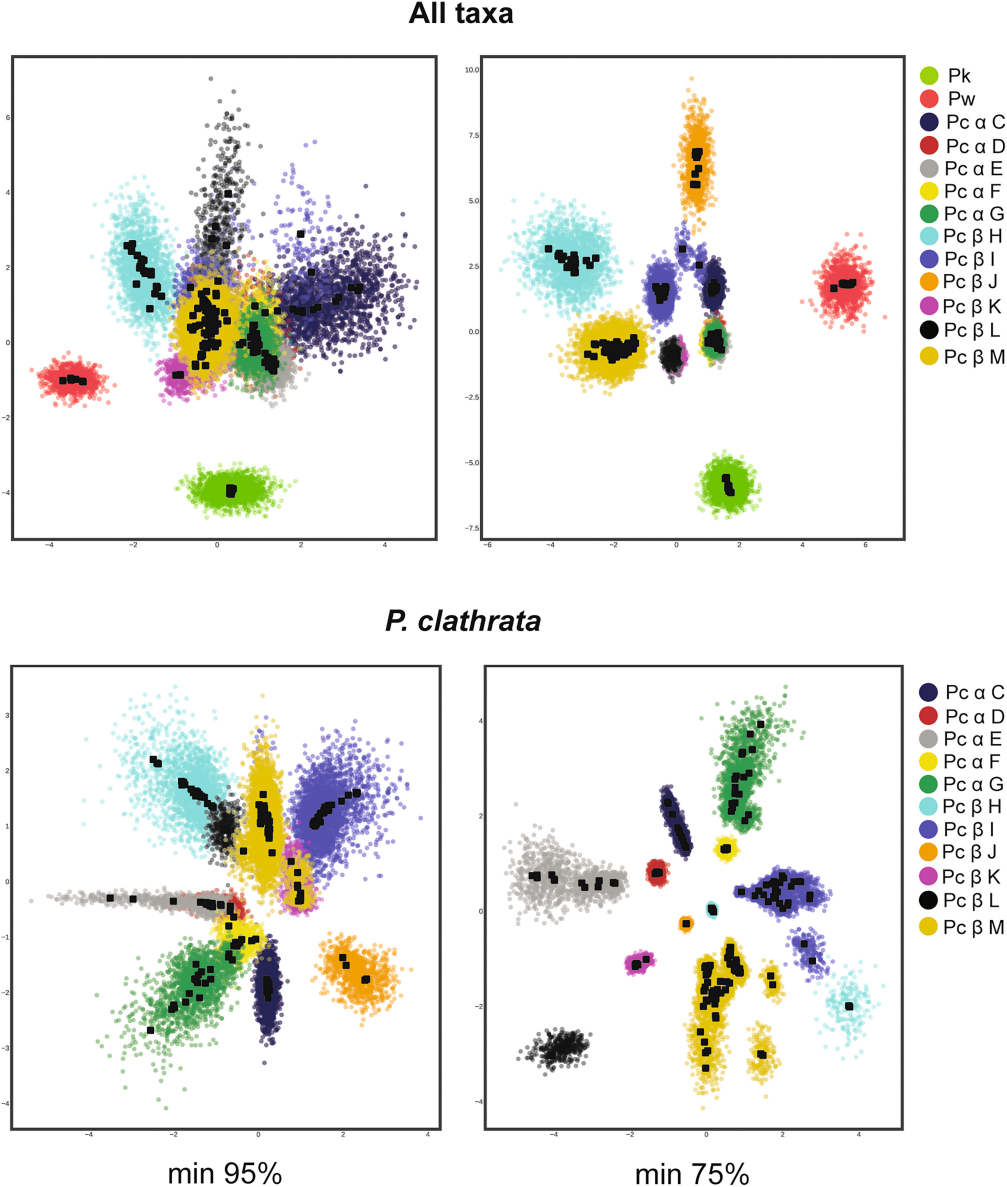
Variational autoencoder cluster analyses of unlinked SNP data. Top panes show analysis with all species. Bottom panes show analysis of *Promyrmekiaphila clathrata* phylogeographic groups only. Left and right panes are based on minimum of 95% and 75% locus occupancy, respectively. Black open circles represent the mean and color clouds represent the standard deviation for the latent distribution. Group colors are consistent with Figure 1.

Variational auto encoders analyses with all data formed three main clusters across all minimum locus thresholds and random SNP replicates (Figure 3; Figure S4). Two of these clusters are well‐isolated and consist of representatives of *P. winnemem* and *P. korematsui* sp. nov. The third cluster is relatively diffused in latent distribution and consists of representatives of the 11 hypothesized lineages of *P. clathrata*. The 11 hypothesized lineages cluster into the two main phylogenetic groups recovered within *P. clathrata*, although this clustering occurs more so in datasets with more SNPs (but also more missing data).

For the 95% minimum locus SNP sets, each of the 11 *P. clathrata* lineages have obvious overlap with one or more other lineages with the exception of the relatively isolated lineage H in SNP set 5 (Figure 3; Figure S4). For the 75% minimum locus SNP sets, lineages generally show greater separation compared to the results of the 95% SNP sets analyses. In SNP set 1 lineages H, J, and M each has minimal overlap with other lineages. Lineage J also exhibits minimal overlap in SNP sets 3 and 4, and lineage H is relatively isolated in SNP set 5. In four out of the five SNP sets the two main lineages that make up *P. clathrata* (α and β) are nearly contiguous, exhibiting minimal overlap.

For VAE analyses that include only *P. clathrata* lineages (Figure 3; Figure S5), in the 95% SNP sets, lineage J is isolated in SNP sets 1 and 5, lineage H is isolated in SNP set 2, lineage L is isolated in SNP set 2, lineage C is isolated in SNP set 4, and lineage I is isolated in SNP set 3. In all five SNP sets the two main lineages (α and β) are isolated, but appear close in latent distribution. For all five 75% SNP sets, the individual lineages exhibited minimal to no overlap with each other, and the two main lineages (α and β) are distinctly isolated.

#### 3.3.2 Ancestry coefficients and gene flow

**FIGURE 4:**
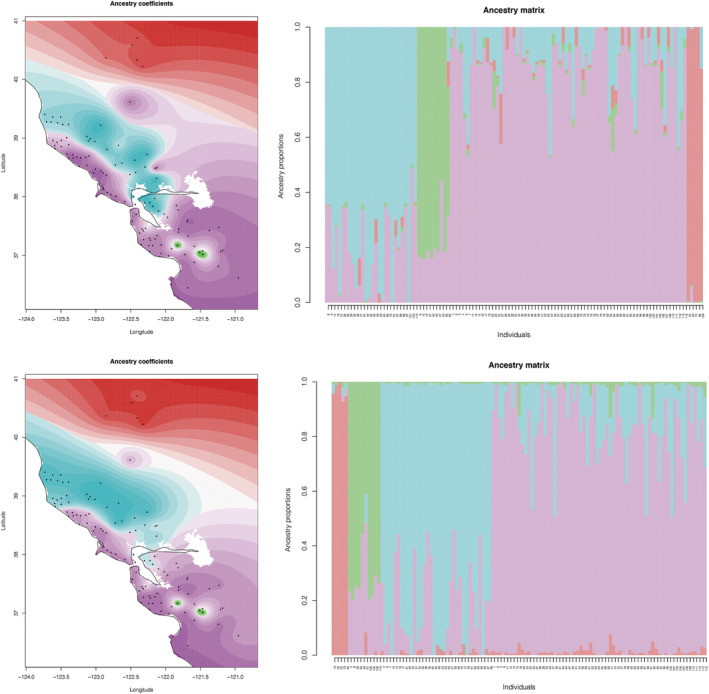
Ancestry coefficient mapping and bar plots for all *Promyrmekiaphila*, based on unlinked SNP data. Top and bottom panes are based on minimum of 95% and 75% locus occupancy data, respectively. Colors are consistent with the four major groups in Figure 1.

For clustering results for all individuals with *K* = 4 and the SNP sets based on minimum 95% locus occupancy, 11%–66% of *P*. *korematsui* sp. nov. individuals were inferred to have ≥0.2 proportion shared ancestry with other groups (mostly with *P*. *clathrata* β clade, group M), and a single individual from *P*. *clathrata* β clade (group M) shared ancestry with *P*. *korematsui* sp. nov. individuals in all five SNP sets (Figure 4; Figure S6). Four out of five SNP sets had no *P*. *winnemem* individuals with ≥0.2 proportion shared ancestry with the other groups. One of the five SNP sets had a single individual (i.e., 20% of individuals) inferred to have ≥0.2 shared ancestry with other groups (mostly *P*. *clathrata* β clade), and five individuals from *P*. *clathrata* α clade (group E) shared most ancestry with *P*. *winnemem* individuals. Twenty‐nine to 50% of *P*. *clathrata* individuals were inferred to have ≥0.2 proportion shared ancestry, but this was mostly shared across *P*. *clathrata* clades. For the SNP sets based on a minimum 75% locus occupancy, 56%–100% of *P*. *korematsui* sp. nov. individuals were inferred to have ≥0.2 proportion of shared ancestry with other groups (mostly with *P*. *clathrata* β clade, group M), and a single individual from *P*. *clathrata* β clade (group M) shared substantial ancestry with *P*. *korematsui* sp. nov. individuals in three of the five SNP sets (Figure 4; Figure S7). No *P*. *winnemem* individuals were inferred to have ≥0.2 proportion of shared ancestry with other groups. 27%–41% of *P*. *clathrata* individuals were inferred to have ≥0.2 shared ancestry, but this was mostly shared across *P*. *clathrata* clades.

**FIGURE 5:**
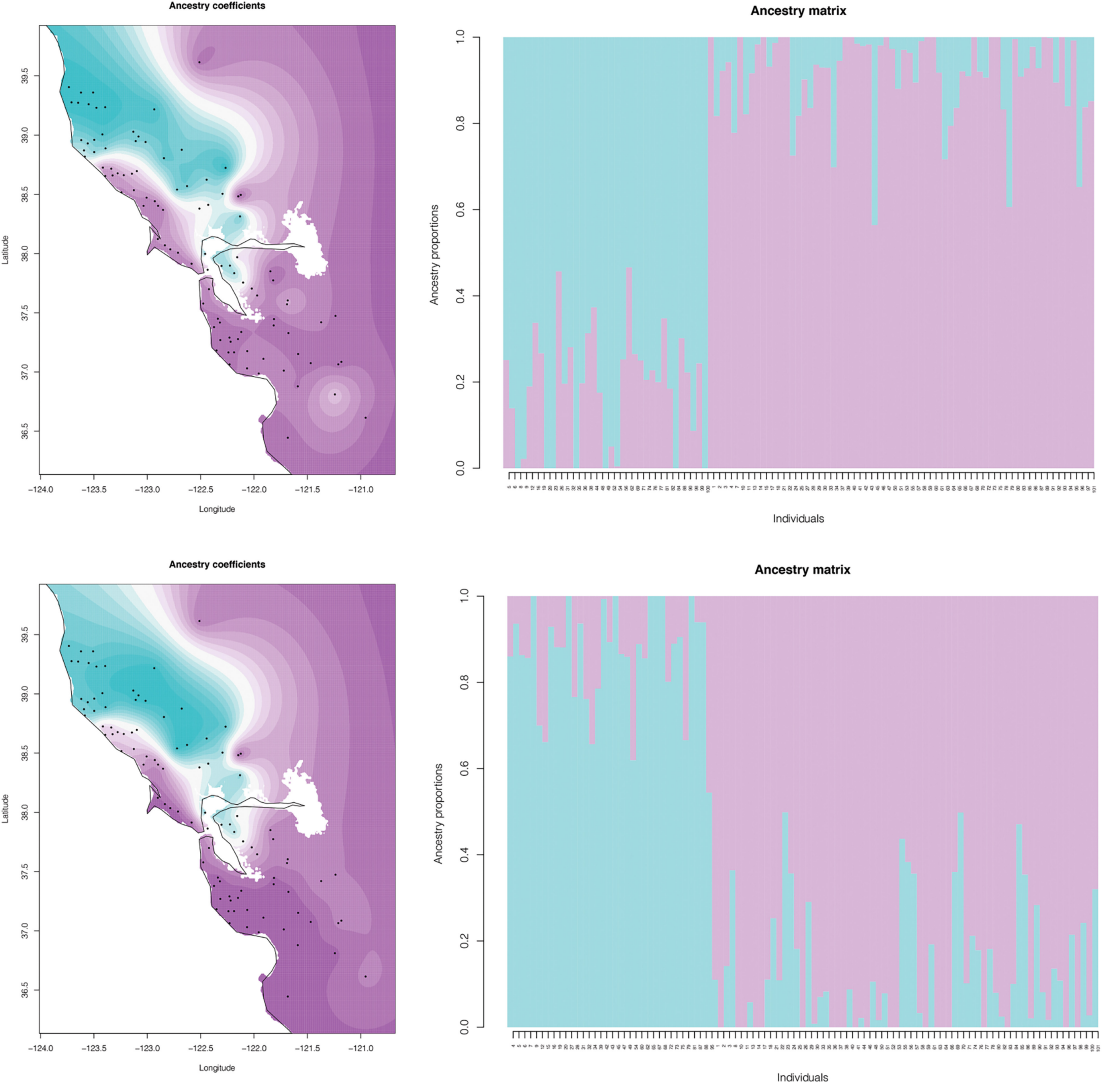
Ancestry coefficient mapping and barplots for *Promyrmekiaphila clathrata* α (turquoise) and β (purple) clades, based on unlinked SNP data. Top and bottom panes are based on minimum of 95% and 75% locus occupancy data, respectively.

For clustering results for *P*. *clathrata* only with *K* = 2 and the SNP sets based on the minimum 95% locus occupancy, 14%–26% of individuals were inferred to have ≥0.2 proportion shared ancestry across *P*. *clathrata* clades (Figure 5; Figure S8). For the SNP sets based on minimum 75% locus occupancy, 26%–44% of individuals were inferred to have ≥0.2 proportion shared ancestry across *P*. *clathrata* clades (Figure 5; Figure S9). For all *P*. *clathrata* only *K* = 11 SNP sets, shared ancestry with one or more other populations occurs for most individuals (not shown).

For the D3 calculation, the average pairwise distances between *P*. *korematsui* sp. nov and *P*. *clathrata* and between *P*. *korematsui* sp. nov and *P*. *winnemem* was 0.0607 and 0.0717, respectively. Full pairwise distance calculations among all individuals and the D3 calculation are available in DRYAD (https://doi.org/10.5061/dryad.4mw6m90gv). The resulting D3 value equaled 0.0829, indicating possible low level of gene flow between *P*. *korematsui* sp. nov and *P*. *clathrata*.

Analysis of both groups of individuals failed to reject the null hypothesis of no gene flow between *P*. *clathrata* α and β. Results of 3‐s runs on the same dataset were similar for most estimated values. We report MLE values from the M2 (isolation with migration) model with the highest likelihood, although for both datasets the model did not fit the data significantly better than M0 (no gene flow). Analysis of group 1 (762 loci) resulted in ln*L* of −90,358.18 (LRT statistic = 0.009) and *θ*_(αβw)_ = 0.020, *θ*_(αβ)_ = 0.003, *τ*_(αβw)_ = 0.006, *τ*_(αβ)_ = 0.003, *θ*_(α)_ = 0.882, *θ*_(β)_ = 0.882, *M*_(αβ)_ = 0.222, *M*_(βα)_ = 0.153. Analysis of group 2 (1061 loci) resulted in ln*L* of −119,439.22 (LRT statistic = 0.008) and *θ*_(αβw)_ = 0.017, *θ*_(αβ)_ = 0.004, *τ*_(αβw)_ = 0.006, *τ*_(αβ)_ = 0.003, *θ*_(α)_ = 0.890, *θ*_(β)_ = 0.890, *M*_(αβ)_ = 0.157, *M*_(βα)_ = 0.142.

#### 3.3.3 CLADES delimitation

Results of species delimitation analysis using CLADES are summarized in Table 1. Analyses were sensitive to input dataset and delimitation model. Generally, the Metano_CLADES model yielded more conservative delimitation results, as expected given the model was generated from a short‐range endemic arachnid group (Derkarabetian et al., 2022). Within the *P*. *clathrata* α clade, the default ALL_CLADES model delimited all five lineages as individual species, but with low probability (.006). The Metano_CLADES model lumped all five lineages into one species, but also had low probability (.036). Within the *P*. *clathrata* β clade, the default ALL_CLADES model delimited the lineages into two species (I + M & H + J + K + L; probability 0.012), but again the Metano_CLADES model lumped all six lineages into one species (probability .055). For the test to determine if the major phylogenetic split within *P*. *clathrata* (α vs. β) was consistent with two species, CLADES lumped both populations into a single species (i.e., *P. clathrata*) regardless of delimitation model with probabilities of .656 and .832 for ALL and Metano models, respectively.

**TABLE 1:** Supervised machine learning (CLADES) species hypothesis test results for *Promyrmekiaphila clathrata*.

Group	Delimitation test groups	Training model	Best assignment of species clusters	Assignment probability	Inferred number of species
*P. clathrata*	α, β	All	(α, β)	.656	1
*P. clathrata*	α, β	Metano	(α, β)	.832	1
*P. clathrata* α	C, D, E, F, G	All	(C), (D), (E), (F), (G)	.006	5
*P. clathrata* α	C, D, E, F, G	Metano	(C, D, E, F, G)	.036	1
*P. clathrata* β	H, I, J, K, L, M	All	(I, M), (K, L, J, H)	.012	2
*P. clathrata* β	H, I, J, K, L, M	Metano	(I, H, M, K, L, J)	.055	1

#### 3.3.4 Morphology

**FIGURE 6:**
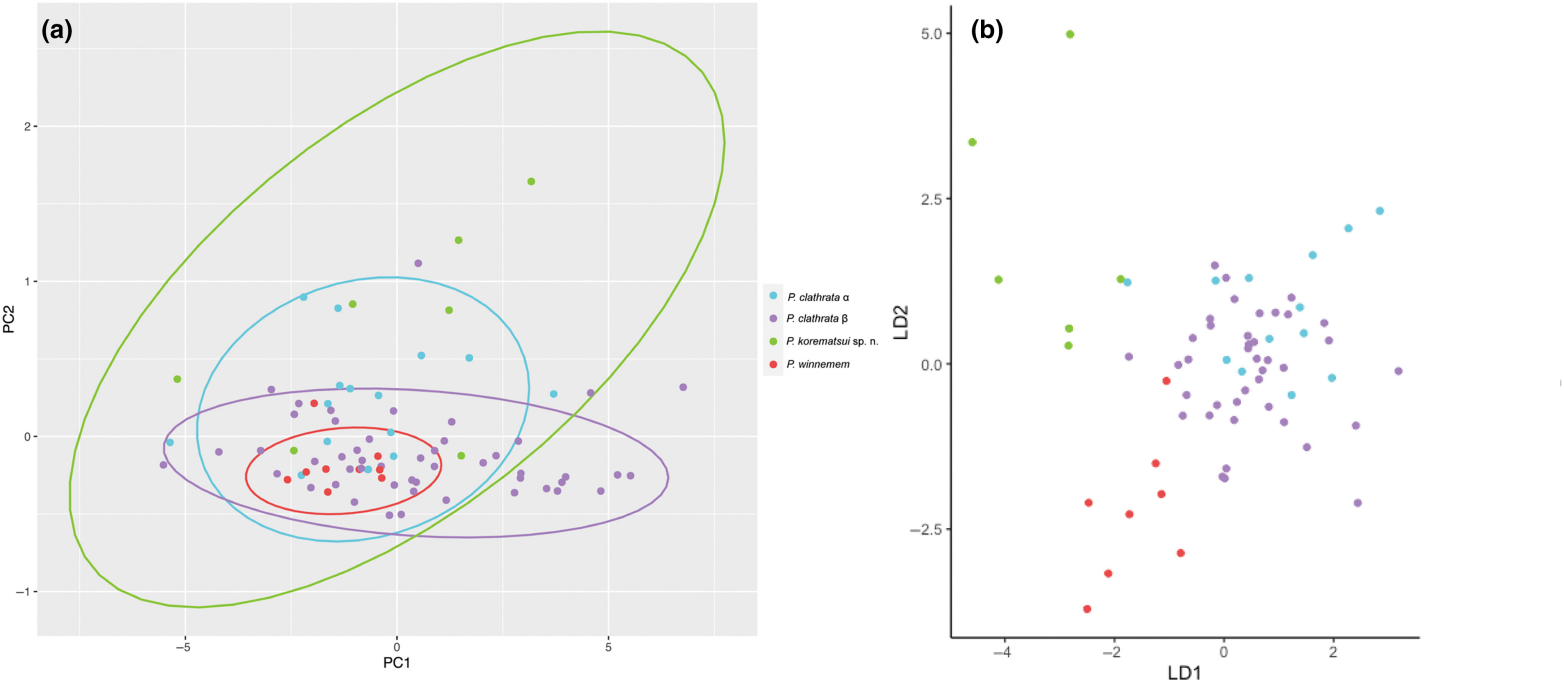
Plot of principal components 1 and 2 (a) and linear discriminant analysis scatterplot (b) for *Promyrmekiaphila* female morphological measurement data. Ellipses in PCA are based on the multivariate *t*‐distribution at 95% confidence level.

For the PCA analysis of continuous morphological traits, PC1, PC2, and PC3 accounted for 0.946, 0.025, and 0.006, respectively, of the total variance. In the morphospace plot based on the PC1 and PC2, the 95% confidence level ellipses for each hypothesized species show significant overlap (Figure 6a). The ellipse for *P. korematsui* sp. nov. was largest, reflecting variability among individuals, as well as potential uncertainty due to smaller sample size. The *P. korematsui* sp. nov. ellipse overlaps with all of the *P*. *winnemem* ellipse, all of the *P. clathrata* α ellipse, and much of the *P. clathrata* β ellipse. The *P*. *winnemem* ellipse occurs within the overlapping region of the *P. clathrata* α and *P. clathrata* β ellipses. The *P. clathrata* α and *P. clathrata* β ellipses exhibit moderate overlap.

A linear discriminant analysis was run with individuals assigned to *P. korematsui* sp. nov., *P. winnemem*, *P. clathrata* α, and *P. clathrata* β, as well as with *P*. *clathrata* assigned as one species. For the four species scenario, the proportion of between‐class variance for linear discriminants 1, 2, and 3 are 0.4714, 0.3279, and 0.2006, respectively. The scatterplot shows that *P. korematsui* sp. nov. and *P. winnemem* do not overlap, and both minimally overlap with *P. clathrata* α and *P. clathrata* β (Figure 6b). *Promyrmekiaphila clathrata* α and *P. clathrata* β largely overlap. Classification percentages from cross validation are presented in Table 2.

**TABLE 2:** Linear discriminant analysis cross validation test results for continuous morphological measurement data.

	Classification result			
	*Promyrmekiaphila korematsui*	*Promyrmekiaphila winnemem*	*Promyrmekiaphila clathrata* α	*Promyrmekiaphila clathrata* β
Data source				
*Promyrmekiaphila korematsui*	**43**	*0*	*14*	*43*
*Promyrmekiaphila winnemem*	*20*	**60**	*0*	*20*
*Promyrmekiaphila clathrata* α	*8*	*0*	**54**	*38*
*Promyrmekiaphila clathrata* β	*2*	*6*	*16*	**76**

*Note*: Correct (bold) and incorrect (italicized) classification percentages are shown.

## 4 DISCUSSION

### 4.1 Phylogenetic relationships

We generated locus and sample rich datasets for *Promyrmekiaphila*, sampled throughout its known range, and discovered a species that had previously gone undetected (Stockman & Bond, 2008). Phylogenetic analyses revealed a divergent lineage that is sister to the *P. winnemem* plus *P. clathrata* lineage (Figure 1; Figures S1 and S2). Discovery of cryptic genetic lineages in mygalomorph species has been well‐documented (e.g., Bond et al., 2001; Brandt et al., 2023; Castalanelli et al., 2014; Hamilton et al., 2014; Hendrixson & Bond, 2005a; Monjaraz‐Ruedas et al., 2023; Newton et al., 2023; Starrett & Hedin, 2007). However, *P. korematsui* sp. nov. is remarkable in that its small distribution is situated well within the distribution of a close relative, the widespread and densely sampled *P. clathrata* (Figure 2).

Sympatry (or very near parapatry) among closely related mygalomorph species is uncommon; rather, most closely related species have allopatric distributions. More distantly related species can be found in sympatry, but the mechanisms for why these species can coexist is not well‐understood in mygalomorphs. Micro‐level differences in niche preference and burrow entrance shape are hypothesized to factor into mygalomorph species syntopy (Bond, 2012; Coyle & Icenogle, 1994; Satler et al., 2011; Wilson et al., 2018). Formal testing of these contact zone dynamics and speciation hypotheses in mygalomorphs is lacking.

The concatenated and coalescent‐based phylogenies generated in our study indicated the presence of at least three species of *Promyrmekiaphila*, and extensive population subdivision within the geographically widespread *P*. *clathrata* (Figure 1; Figures S1 and S2). Phylogenetic relationships in *Promyrmekiaphila* inferred from UCE loci are generally similar to those from the mitochondrial based tree, with *P. winnemem* sister to *P*. *clathrata*, which together form a sister group to *P*. *korematsui* sp. nov. (Figure S3; Stockman & Bond, 2007). Each of these three species is strongly supported across all analyses in the UCE data and with high bootstrap support (>90%) in the ML analysis of the COI data (Figure 1; Figure S3).

Within *P*. *clathrata*, there is strong support for 11 distinct lineages across phylogenetic analyses of UCE data (Figure 1). Nine of the 11 lineages within *P*. *clathrata* that have strong support with UCE data (100% ASTRAL BS; Figure S2) were recovered in the mitochondrial tree as well. However, in the COI tree, *P*. *clathrata* α is paraphyletic with respect to the *P*. *clathrata* β clade (Figure S3). Incongruences in relationships among lineages that are evident across the UCE (Figure S2) and COI datasets are generally associated with nodes receiving low support (COI BS <90%). The uncertainty in relationships among lineages within the two major *P*. *clathrata* clades, indicated by polytomies toward the root of each group (Figure S2), not only likely reflects gene tree‐species tree conflict due to insufficient time for lineage sorting but also may indicate inadequate phylogenetic information in individual gene trees. Lineage sorting would require even greater time in a large population that underwent multiple fragmentation events that occurred close together in time (Maddison, 1997). Ongoing gene flow may also be occurring, which could lead to gene tree‐species tree incongruence. However, mitochondrial discordance associated with high nodal support, which could indicate recent introgression, was not evident (Aguillon et al., 2022; Funk & Omland, 2003). Additional genomic‐based studies examining population divergence within *P*. *clathrata* may shed light on how the dynamic geology and ecology of the San Francisco Bay Area has shaped biodiversity in this region.

### 4.2 Speciation dynamics

Mechanisms of reproductive isolation in mygalomorph spiders are not well understood. Upon reaching maturity, males emerge from their burrows and seek females, likely by detecting pheromones associated with the female's silk‐lined burrow (Copperi et al., 2019; Costa et al., 2013; Coyle, 1985). The male may tap on the burrow entrance to generate vibratory signals to entice the female to the burrow entrance to mate (Costa et al., 2013; Coyle & Shear, 1981; Ferretti et al., 2013; Frank et al., 2023; Jackson & Pollard, 1990). Reproductive isolation may be maintained between closely related species that geographically overlap by prezygotic mechanisms, such as differences in pheromone chemical composition or vibrational cues, incompatibility of traits involved in copulation, or a combination of these factors. Selection against sub‐optimal interspecific mating between sympatric species would be expected to result in mechanisms that reinforce prezygotic isolation (e.g., species specific recognition (Costa et al., 2013), courtship behavior (Frank et al., 2023; Postiglioni & Costa, 2006), maturation time (Hendrixson & Bond, 2005b)).

*Promyrmekiaphila korematsui* sp. nov. likely occurs in sympatry with *P. clathrata*, which is comparatively widespread (Figure 2). Although we did not find syntopy between *P. korematsui* sp. nov. and *P. clathrata* (i.e., individuals of each species have not been obtained from burrows occurring at the same collection site), we predict that denser sampling of localities and individuals will reveal areas where these species overlap given their proximity and the absence of obvious barriers to migration. Consequently, *P. korematsui* sp. nov. and *P. clathrata* likely come into contact, possibly frequently, providing ample opportunities for gene flow between these species. No discernable ecological barriers to gene flow separate these two species, but *P*. *korematsui* sp. nov. is currently known from only five localities, making analyses of ecological differentiation unreliable (e.g., niche model analyses, see Newton et al., 2023). Yet, *P. korematsui* sp. nov. forms a distinct genetic cluster that never overlaps with those of *P. clathrata* and *P. winnemem* in VAE analysis of any SNP set (Figure 3; Figure S4), indicating that a long duration of reproductive isolation has occurred.

*Promyrmekiaphila korematsui* sp. nov. is distributed near the San Andreas Fault in the San Francisco Peninsula region (Figure 2). This region has undergone considerable geologic change, for example, repeated flooding of the Pajaro River and movement along the San Andreas Fault, potentially causing barriers to gene flow contributing to speciation in low‐vagility taxa (Bennett et al., 2019; Bond & Stockman, 2008; Briggs & Ubick, 1989; Gottscho, 2016; Hedin et al., 2013; Kuchta et al., 2009; Martínez‐Solano & Lawson, 2009; Wake, 1997). In Santa Clara County, where we find *P. clathrata* and *P. korematsui* sp. nov. in near sympatry today, the San Andreas and Calaveras faults are still active. These faults have known movement from the Holocene and Pleistocene Epochs (Harden, 2004); thus, plate tectonics may have been a contributing factor to the resulting overlapping distributions of these species. More recently established barriers to gene flow are represented by the dry Santa Clara Valley where, despite extensive sampling efforts, no *Promyrmekiaphila* have been collected, and the San Francisco Bay, which has been flooded for the last 10,000 years (Harden, 2004; Stockman & Bond, 2007).

Ancestry coefficients indicate that *P*. *korematsui* sp. nov. and *P. clathrata* experience gene flow or share ancestral polymorphisms (i.e., incomplete lineage sorting; Figure 4; Figures S6 and S7). Shared ancestry could also be overestimated in this case due to use of the default value for the regularization parameter lambda in Tess3, which controls how geographic proximity impacts ancestry estimates (Caye et al., 2016). A more conservative lambda value may be more appropriate for low vagility taxa, but selection of this value is subjective. The D3 statistic also indicates that if gene flow is occurring, it is infrequent. If frequent gene flow occurs between *P. korematsui* sp. nov. and *P. clathrata*, it would be expected to have a homogenizing effect in the absence of natural selection (Slatkin, 1987); however, we find that these two species exhibit some apparent, yet subtle, degree of morphological divergence, both in male secondary sexual characteristics (Figure 8) and somatic characteristics in females (Figure 6). Such differences indicate that selection against hybridization is potentially strong. Although, given the highly restricted geographic range of *P. korematsui* sp. nov., these morphological differences could also be the result of stochastic causes (e.g., population bottleneck). Whether gene flow between *P. korematsui* sp. nov. and *P. clathrata* is restricted due to morphological incompatibility or through other mechanisms is currently unknown, but identifying areas of contact between these species could shed light on speciation mechanisms in morphologically conserved species.

*Promyrmekiaphila winnemem* is allopatric with respect to both *P. korematsui* sp. nov. and *P*. *clathrata*, although areas of contact are possible on the eastern side of the northern coastal range (Figure 2). *Promyrmekiaphila winnemem* forms a distinct and isolated genetic cluster in the VAE analysis (Figure 3; Figure S4), and shows minimal evidence of shared ancestry with its congeners (Figure 4; Figures S6 and S7). Geographic isolation and ecological niche divergence may prevent genetic exchange between *P. winnemem* and *P. clathrata* (Stockman & Bond, 2007). Given the close sister relationship between *P. winnemem* and *P. clathrata*, evidence of incomplete lineage sorting would be expected, but we find minimal evidence of shared ancestry between these species. Similar to *P. korematsui* sp. nov., *P. winnemem* is highly restricted in its geographic range, and shared ancestral polymorphism may have been lost due to a population bottleneck or from a founder event. *Promyrmekiaphila winnemem* and *P. clathrata* exhibit male secondary sexual and female genitalic differences (Stockman & Bond, 2008), but the distribution of variance for somatic continuous characters for *P*. *winnemem* occurs within that of *P*. *clathrata* (Figure 6a). This lack of somatic morphological divergence in females suggests prezygotic mechanisms of selection against hybridization remain weak. Selective pressure for prezygotic isolating mechanisms is likely less severe for allopatric species than for species found in sympatry, where mating could result in low fitness offspring (Servedio & Noor, 2003). In allopatry, postzygotic incompatibility may build up through other mechanisms, such as Dobzhansky–Muller incompatibilities from genetic drift, or hybrid inviability or sterility resulting from chromosome incompatibility (Forejt & Jansa, 2023; Orr & Turelli, 2001).

The deep phylogenetic split (divergence) within *P. clathrata* indicates a long‐standing barrier to gene flow occurred in the history of this species, and may still be acting. The two major population groups (α and β) are distributed in parapatry, and obvious geographic barriers are lacking (Figures 1 and 2). Despite this deep phylogenetic divergence, the genetic clusters from these groups come into close contact in the VAE analyses, and partially overlap, in the case of some SNP sets (Figure 3; Figures S4 and S5). Numerous individuals show evidence of shared ancestry across populations, which could reflect ongoing gene flow or incomplete lineage sorting (Figure 5; Figures S8 and S9), although statistical support was lacking for the gene flow model (M2) in the 3s analysis. Given the influence of geography on estimation of ancestry coefficients with Tess3, it is not surprising that individuals generally in areas of close contact between the major populations exhibit mixed ancestry and population assignment ambiguity. These individuals are mostly distributed north of the Bay, along the coast as well as eastern edge of the Coast Range, in mountain ranges that run parallel with the coast (Figure 2). Although *P*. *clathrata* populations are not currently strictly separated into different mountain ranges, orogeny processes and their timing likely have had an important role in population subdivision and contact dynamics in this species and other low‐vagility species in this region (Bryson et al., 2016; Hedin et al., 2013; Howard, 1979; Kuchta et al., 2009; Leavitt et al., 2015).

*Promyrmekiaphila clathrata* clades α and β show a high degree of overlap in the female morphological character PCA (Figure 6a), which indicates gene flow could be limiting morphological divergence. Alternatively, α and β populations may be incipient species, and insufficient time has occurred for selection to produce significant morphological differences. Selection against hybridization where these parapatric lineages potentially come into contact might explain non‐overlapping data points in the PCA. No obvious differences in genitalic and somatic morphology occur for males from the two main populations of *P*. *clathrata*, but limited sample size prevents more formal analyses (Stockman & Bond, 2008). Establishing the structure and divergence levels within *P*. *clathrata* will facilitate a comparative approach to testing possible ecological and behavioral reproductive barriers (Bond & Stockman, 2008; Chambers et al., 2023; Garrison et al., 2020; Masta & Maddison, 2002; Newton et al., 2020; Starrett et al., 2018).

Population subdivision within *P*. *clathrata* α and β clades is evident from the phylogenetic analyses (i.e., clades C–M; Figure 1; Figure S1). By using monophyletic groups found in the strict consensus of the MSC ASTRAL bootstrap analysis (Figure S2), we applied a highly conservative threshold for defining lineages. Yet, these groups do not consistently separate in the clustering and ancestry coefficient analyses with different SNP sets (Figure 3). Species delimitation assessments using SNP data within *P*. *clathrata* were sensitive to the threshold of locus occupancy, different random SNP sets, and the inclusion of its congeners. Different subpopulations within *P*. *clathrata* formed an isolated cluster in VAE analysis of some, but not all, random SNP sets (Figures S4 and S5). Inconsistencies in clustering could be due to SNPs having different genetic histories due to recombination, stochasticity in levels of information content in SNP sets, poor model fit of nucleotide substitutions at SNP sites, or a combination of these factors. Although machine learning clustering methods using SNP data have been shown to have utility in determining population‐level evolution as well as in delimitating population‐species boundaries (Battey et al., 2021; Derkarabetian et al., 2019; Newton et al., 2020, 2023), analysis of a single random SNP set for species delimitation could produce misleading results, particularly if the SNP sample size is limited.

Species delimitation of *P*. *clathrata* based on the supervised machine learning analysis (CLADES) with the low‐vagility arachnid group (*Metanonychus*) training model also supports the single species hypothesis (Table 1). Delimitation with the taxonomically broad default training model (All) gave inconclusive results, not only supporting delimitation of some groups within the two major populations as species but also not supporting the two major populations into distinct species. Expansion of the default training model to include more low‐vagility species, or development of a new training model based on taxa more closely related and ecologically similar to *Promyrmekiaphila*, could make this delimitation method more informative for mygalomorph spiders (Derkarabetian et al., 2022; Newton et al., 2023).

Despite extensive genomic and population level sampling, questions regarding cryptic species delimitation in *Promyrmekiaphila* remain unresolved. Using an integrative approach that evaluates both genetic and demographic exchangeability (i.e., the Cohesion Species Concept, see Newton et al., 2023), we determine through a process of reciprocal illumination that the lineage comprising *P. korematsui* sp. nov. populations is genetically divergent and isolated, although infrequent gene flow may occur. We find subtle phenotypic differences that are suggestive of character displacement or reinforcement where *P. clathrata and P. korematsui* sp. nov. potentially share a zone of direct contact. Although delimitation of *P. korematsui* sp. nov. appears relatively straightforward, employing a similarly integrative framework for lineages embedded within *P. clathrata* leaves more questions than definitive answers. Apparent gene flow, that may have occurred recently, and phenotypic cohesion across populations fails to solidly reject hypotheses of genetic and demographic exchangeability and thus we retain *P. clathrata* as a single species. Additional population sampling and acquisition of more male specimens where divergent lineages come in to contact (e.g., α/β) is needed to fully resolve species delimitation within this species.

### 4.3 Taxonomy (Figures 7 and 8)

**FIGURE 7:**
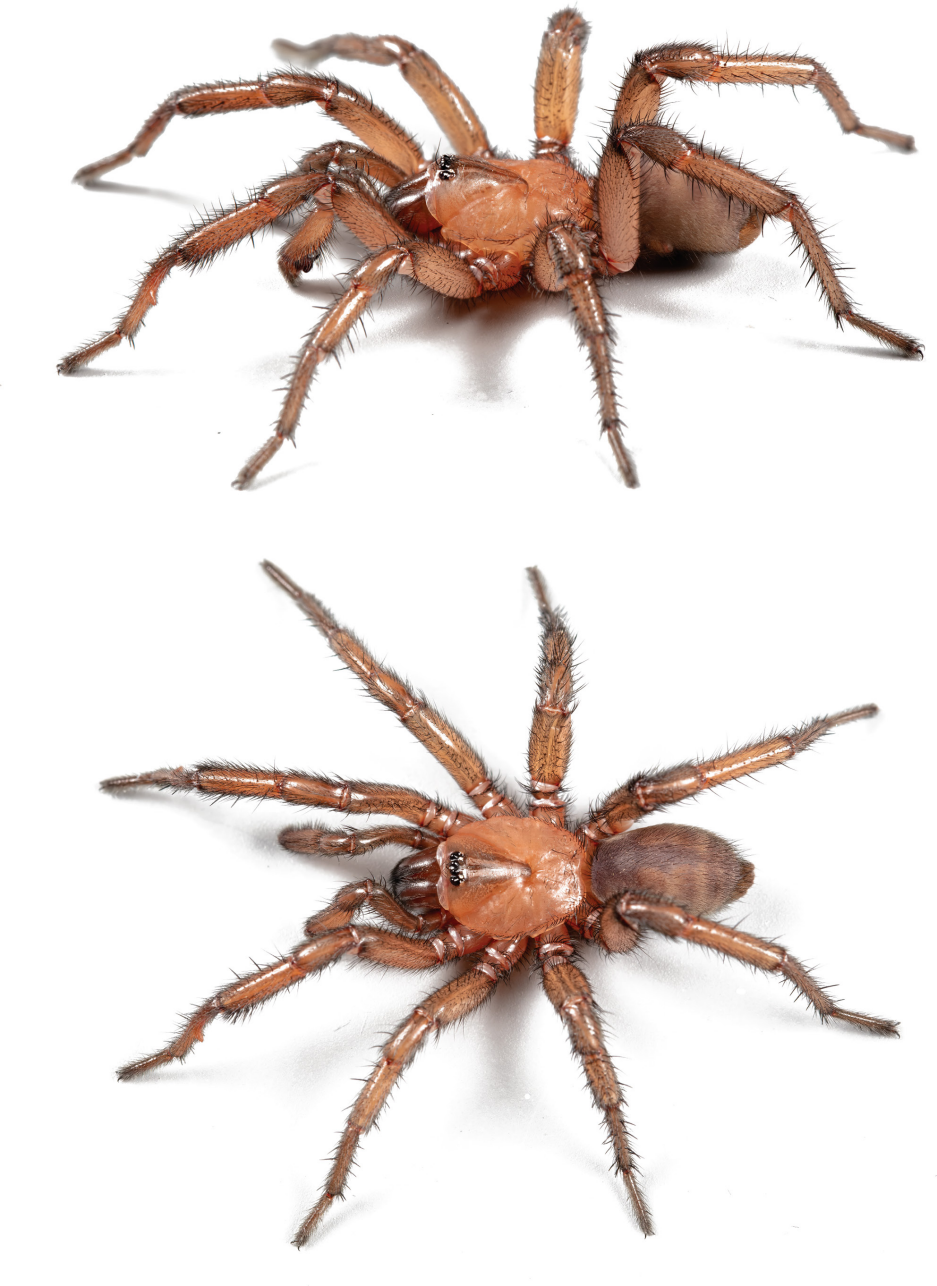
*Promyrmekiaphila korematsui* sp. nov. HOLOTYPE male live habitus photographs, side and dorsal views.

**FIGURE 8:**
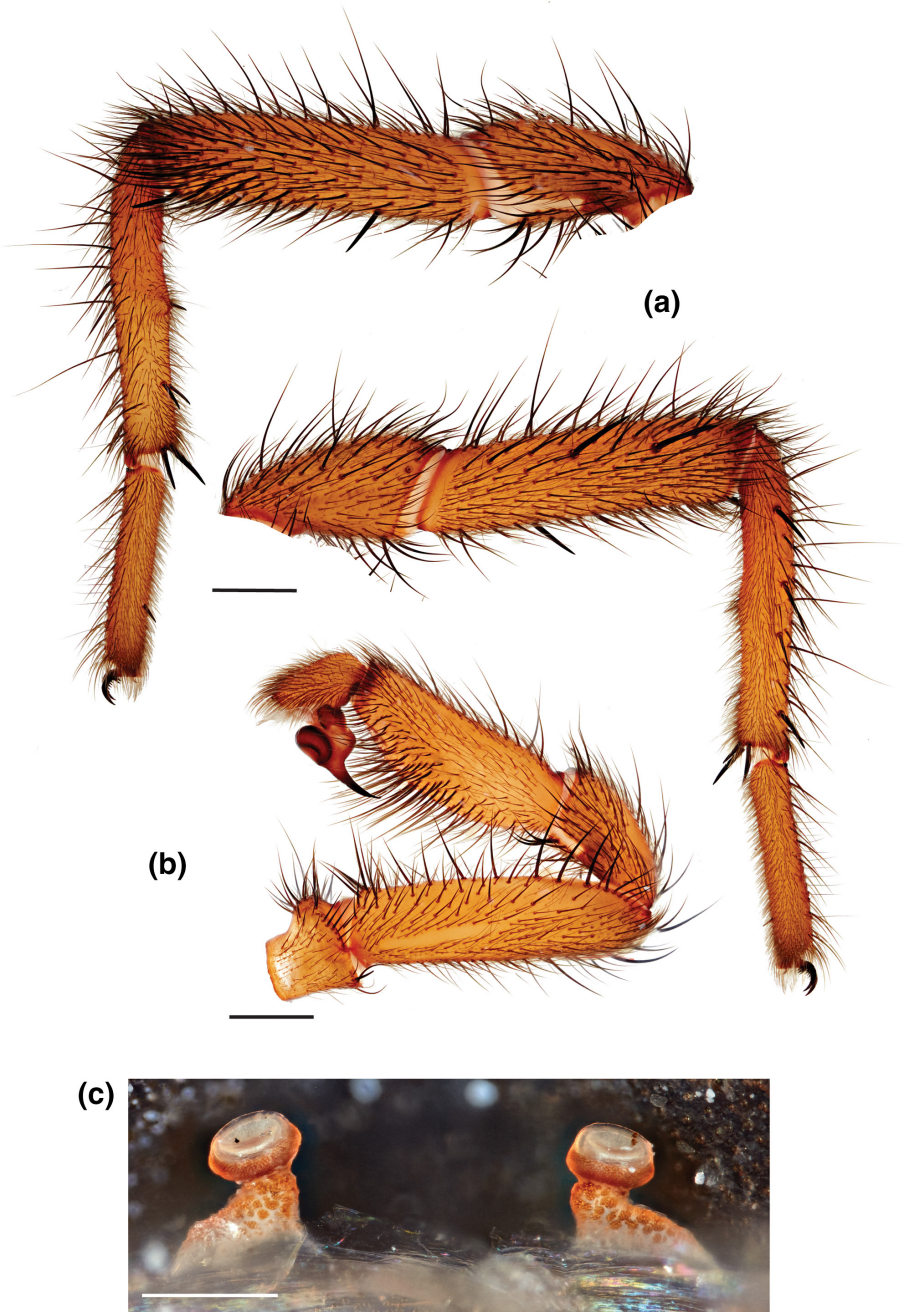
*Promyrmekiaphila korematsui* sp. nov. (a, b) HOLOTYPE male. (a) retrolateral and prolateral views of leg I, (b) retrolateral view of pedipalp; scale bars – 1 mm. (c) PARATYPE female spermathecae; scale bar = 0.25 mm.

Family Euctenizidae Raven 1985

Subfamily Euctenizinae Raven 1985

Type Genus: *Eucteniza* Ausserer 1875

Included Euctenizinae Genera: *Entychides* Simon, 1888; *Eucteniza* Ausserer, 1875; Neoapachella Bond and Opell, 2002; *Promyrmekiaphila* Schenkel, 1950; *Cryptocteniza*, Bond and Hamilton 2020

*Promyrmekiaphila* Schenkel, 1950, (type species by monotypy *Promyrmekiaphila gertschi* Schenkel)

*Promyrmekiaphila korematsui* sp. nov. Bond, Jochim, Quayle, and Starrett

urn:lsid:zoobank.org:act:CD07772A‐1EE2‐4906‐BB4B‐2A343F82A97D

#### 4.3.1 Diagnosis

This species is very similar to *P. clathrata*; males have a ventral‐to‐retrolateral excavation on the proximal third of the metatarsus (Figure 8a); however, the apophysis is more slender and elongate. *Promyrmekiaphila korematsui* has fewer distal spines on the retrolateral surface of the tibia I mating clasper than *P. clathrata* and has 4–5 distinct spines along the centerline of the ventral surface of metatarsus I; both of the other species lack these spines.

#### 4.3.2 Etymology

The specific epithet is a patronym in honor of Fred Toyosaburo Korematsu. Korematsu was awarded the United States Presidential Medal of Freedom in 1998 in recognition of his lifelong dedication as a civil rights activist and his resistance to the incarceration of Japanese Americans in concentration camps during World War II.

#### 4.3.3 Type material

HOLOTYPE MALE (BMEA0102984; deposited in the BME) and PARATYPE MALE (BMEA0102982; deposited in the CAS) from United States, California, San Mateo Co. Clarkia Trail in Edgewood Park, N 37.45935 W −122.28546, coll. By E. Jochim, L. Chamberland, J. Starrett, 2.x.2021. Two PARATYPE FEMALES (MY3285, 3286; deposited in the BME) from United States, California, Santa Clara Co., Leavesley Rd., 0.9 mil S of jct w/Roop Rd., N 37.05034 W −121.51101, 190 m, coll. By A. Stockman, 7.vi.2005.

MALE HOLOTYPE. *Specimen preparation and condition*. Specimen preserved in 70% EtOH. Pedipalp, leg I removed, stored in vial with specimen. *General coloration in alcohol*. Carapace strong brown 7.5YR 5/6; abdomen lighter with wide dusky bands (Figure 8). *Cephalothorax*. Carapace 5.82 long, 5.44 wide, setose, pars cephalica elevated. Fringe with heavy setae extending to midline. Thoracic fovea groove deep, procurved. Eyes on slightly raised tubercle. AER slight procurve, PER slight recurve. PME, AME, subequal diameter. Sternum moderately setose, STRl 3.22, STRw 3.06. Posterior sternal sigilla large, oval, not contiguous; anterior sigilla pair smaller, at margin. ANTd comprising 8 large teeth; posterior margin with patch of ~14 smaller teeth. Palpal coxa, numerous cuspules across entire surface, labium lacks cuspules, LBw 1.24, LBl 0.69. Rastellum 3–4 stout spines not on a mound. Abdomen. Moderately setose; apical segment of PLS short, triangular in shape. Legs. Leg I: 5.40, 2.83, 4.20, 3.85, 2.42; leg IV: 6.08, 2.83, 4.91, 4.71, 2.59. Moderately dense scopulae on tarsi I, II and distal half of metatarsi I, II. Tarsus I with thin band of ~12 trichobothria. ITC small, gently curved. Leg I spination pattern (Figure 8a); TSp 2, TSr 0, TSrd 2. Pedipalp. PTw 1.02, PTl 2.60, Bl 1.22. Embolus arises sharply from copulatory bulb, thin taper distally (Figure 8b).

Variation. Males known only from the type specimens.

FEMALE PARATYPE (MY3285). Specimen preparation and condition. Specimen preserved in same manner as male holotype. Color. Same as male. Cephalothorax. Carapace 6.94 long, 6.31 wide, glabrous. Lacks fringe. Thoracic fovea groove deep and procurved. Tubercle absent. PER nearly straight, AER straight to slightly procurved. AME, PME subequal diameter. Sternum moderately setose, STRl 4.42, STRw 3.83. Posterior sigilla large, widely separated sub‐oval in shape; medial anterior sigilla relatively small, positioned laterally. ANTd with ~8 teeth with posterior margin comprising denticle patch. Palpal coxae, numerous cuspules, spread evenly across; labium lacks cuspules, LBw 1.68, LBl 1.01. Rastellum comprises 4 short spines not on a tubercle. Legs. Leg I: 4.92, 3.03, 3.45, 2.26, 1.29; leg IV: 6.05, 3.37, 4.47, 3.96, 1.99. Dense scopulae tarsus/metatarsus of Legs I/II, tarsus/tibia of pedipalp. Tarsus I with ~12 trichobothria arranged in a relatively tight staggered row. PTLs 10, TBs 6. ITC small, gently curved. Preening combs on leg IV. Anterior spermathecae with wide base but short, unbranched stalk (Figure 8c). Apical segment of PLS short, domed.

Variation (*n* = 8). Cl 4.03–7.95, 6.22 ± 0.49; Cw 3.79–7.43, 5.72 ± 0.46; STRl 2.6–4.86, 3.83 ± 0.3; STRw 2.4–4.41, 3.48 ± 0.25; LBw 1.01–1.89, 1.32 ± 0.13; LBl 0.62–1.22, 0.9 ± 0.08; Leg I 19.38–17.32, 14.25 ± 1.01; Leg IV 11.42–20.89, 17.53 ± 1.29; TBs 3–6, 3.57 ± 0.43.

Additional material examined. UNITED STATES: CALIFORNIA: **San Benito Co**: Lone Tree Rd, NE of Hollister [36.91560 −121.27340, MY4016], 27.iii.2011 (M. Hedin, J. Starrett, D. Leavitt, J. Satler, D. Carlson, B. Keith), 1 female; McCreery Ranch Rd, E of Paicines [36.76850 −121.19280, MY3798], 27.iii.2011 (M. Hedin, J. Starrett, D. Leavitt, J. Satler, D. Carlson, B. Keith), 1 female. **San Mateo Co**: Clarkia Trail in Edgewood Park [37.45935 −122.28546, BME102981, BME102986], 2.x.2021 (E. Jochim, L. Chamberland, J. Starrett), 2 imm; Edgewood Park, Clarkia Trail, off Cañada Rd., near Hwy 280, E of San Carlos [37.45935 −122.28546, MY3537], 12.iii.2006 (M. Hedin, R. Keith, S. Thomas, J. Starrett), 1 imm. **Santa Clara Co**: Almaden Rd, 1.0 mi E of Hicks Rd [37.16645 −121.82911, MY3333, MY3334, MY3335], 11.vi.2005 (A. Stockman), 3 imm; Canada Rd, SW of Jamieson Rd [37.01569 −121.49001, MY3468, MY3469, MY3470, MY3471], 24.i.2006 (A. Stockman, P. Marek), 4 females.

Distribution. California: Central California in the San Francisco Bay Area. Proximate to the San Andreas Fault line, from mid‐San Francisco Peninsula to the Diablo Range, east of Paicines.

Natural history. Found in shaded ravines and along roadcuts in oak woodland habitat. Most males likely mature in early Autumn.

Conservation status. Using NatureServe (Faber‐Langendoen et al., 2012) Conservation Status Rank criteria, we consider the status of *P. korematsui* to be CRITICALLY IMPERILED because of its high risk of extinction due to having a very restricted range of only a few geographical close localities.

## AUTHOR CONTRIBUTIONS

**James Starrett:** Conceptualization (equal); formal analysis (lead); funding acquisition (supporting); investigation (lead); methodology (equal); supervision (lead); validation (lead); visualization (equal); writing – original draft (equal); writing – review and editing (equal). **Emma E. Jochim:** Formal analysis (supporting); investigation (supporting); visualization (equal); writing – original draft (supporting); writing – review and editing (equal). **Iris L. Quayle:** Investigation (supporting); writing – review and editing (equal). **Xavier J. Zahnle:** Investigation (supporting); writing – review and editing (equal). **Jason E. Bond:** Conceptualization (equal); funding acquisition (lead); investigation (supporting); methodology (equal); project administration (lead); resources (lead); visualization (equal); writing – original draft (equal); writing – review and editing (equal).

## CONFLICT OF INTEREST STATEMENT

The authors declare that they have no conflicts of interest.

## Supporting information


Appendix S1

## Data Availability

Detailed information regarding sequencing results, assemblies, scaffold match, and alignment are provided in DRYAD (Starrett et al., 2024: https://doi.org/10.5061/dryad.4mw6m90gv). Demultiplexed raw reads are available in SRA database (PRJNA1019074). Assembled contigs, sequence alignments, SNP sets, phylogenetic trees, pairwise genetic distances, morphological measurement data, and supplementary figures are available in DRYAD (https://doi.org/10.5061/dryad.4mw6m90gv).
